# Supplementary material for: Patterns of Intron Gain and Loss in Fungi
Source: PLoS Biol. 2004 Nov 30;2(12):e422. doi: 10.1371/journal.pbio.0020422 (PMC532390; doi:10.1371/journal.pbio.0020422)
Supplement: Table S1 — Also available at http://genes.mit.edu/NielsenEtAl/. (4.3 MB ZIP). [file pbio.0020422.st001.zip › NielsenEtAl/html/507.html]

AN6087.1.NCU07478.1.MG03631.1.FG09323.1


```
 CLUSTAL W (1.82) Multiple Sequence Alignments - Introns Inserted


Sequence 1: NCU07478.1	488 aa
Sequence 2: MG03631.1	463 aa
Sequence 3: FG09323.1	476 aa
Sequence 4: AN6087.1	426 aa
Alignment Length: 505 aa
Number Identitical Residues: 168 aa
Alignment Score (without introns) 8659


MG03631.1 	MASLASAPSS-----QIIHNPADHENGTKSITLEKMV-SGAEQAAPLP--LPTGVAGGLS
NCU07478.1	MASILQG----------AHSSASHDFDRIIDHRHDSS-SSASSASPTPSAFPT-VAQDNT
FG09323.1 	MATIGTNRPGGYDHHHHHQNPLQNDDEFSLRHMMDAEPNSPDNSRDVLSGSSSIPATGDG
AN6087.1  	--------------------------------------MAATSGTGSVSTVEASPAG---
          	                                       .. ..    :   :  *    

MG03631.1 	AGDEPVEITAVQKMVSAISGSLLTGLLV1T----PLDVVRVRLQSQTIAKPESSVKAY--
NCU07478.1	APNEPVEITAAQKMLSATSGSLLTGLLV~T----PLDVVRVRWQSQSFTRPSPTAHTVTD
FG09323.1 	KGNSAVEITATQKMVSAMSGSLLTSLLV1SRSVTPLDVVRVRLQSQ---APRSTVDFS--
AN6087.1  	------EVSITQKMVSATCGSVLTGLLV1T----PLDVVRVRLQSQSPIKNTSPFTSH--
          	      *:: .***:** .**:**.*** :    ******** ***:     ..      

MG03631.1 	--KLP--LTSTSAFRPPANIGVTACCREVFFMNNNAQFCVVGG-HTEPSSSRAAALECAV
NCU07478.1	FAKLAGSQISNPAFR-PANLGVTACCREVFFANNSEGCIVATGPRIGSGAITASAIPCAV
FG09323.1 	--KLAITTSSLSPAQ-TAELGITSCCREVFFSGGNTEFCLAAPRIDGITSAPPAPAECAV
AN6087.1  	--------TTPTLKNAPPNLGVTACCREVFWGGQNSEICMVGPSGSALGATSQSVAECAV
          	         : .  ....::*:*:******: . .    :.        :   :   ***

MG03631.1 	EQTQQRTYNSTIDGLRKIARNEGATALWRGLSPTLLMAVPGNIIYFTGYEWLRFNRESPI
NCU07478.1	EETKQRNFNSTLDGLRKIARNEGFTSLWRGLSPTLLMAIPANIIYFTGYEWLRFNPSSPI
FG09323.1 	EEVQRRTFSSTFDGLRKIARNEGVATLWRGLSPTLVMAVPSNIIYFTGYDYLRFNSNSPF
AN6087.1  	EETQRRTFTSTLDGLRKIARNEGVLTLWRGLSPTLMMGIPGNVIYFAGYDWLRTDDRSPI
          	*:.::*.:.**:***********  :*********:*.:*.*:***:**::** :  **:

MG03631.1 	VKVVKEDYSPLVAGSVARILAAGAVSPIELFRTRLQASHGSSAAGHLADTFRGIREMVGL
NCU07478.1	QQTVKEEYAALVAGAGARILAATAVGPIELFRTRMQASPGSTTGSHLTHTFRGIKDMVYA
FG09323.1 	SR-FSDTSAPLTAGSAARILAATAVSPIELVKTRMQAASGASTTNHLVEAFDSVKGMVGT
AN6087.1  	KRVVPGAYVPFVAGAVARVAAATAISPIEMFRTRLQATPG-TGAGHFKATLEGLYQMTQA
          	 : .     .:.**: **: ** *:.***:.:**:**: * :  .*:  :: .:  *.  

MG03631.1 	QGYRSLWRGLTLTLWRDVPFSGMYWWGYEFIRGNLTDARE---RGRGQTRDAD-ESRGRA
NCU07478.1	HGYRSLWRGLTLTLWRDVPFSGMYWWGYETIRGKLTDAREARSRGRGRTLDLDSEERHRV
FG09323.1 	HGYTALWRGLTLTLWRDVPFSGLYWWGYESIRSRLTDYRE---QRQGSTLPFE-DELVEA
AN6087.1  	KGYGSLWRGFTLTMWRDVPFSGLYWWGYEEVKKSIIEVRK---KARGHNPPQT----APS
          	:** :****:***:********:****** ::  : : *:   : :* .           

MG03631.1 	RRRSQSRENHAETFVDSFVAGASSGAFASVATMPFDVGKTRTQVYQSNGGLSKSAK----
NCU07478.1	RRRSQSRENHAETFTDSFIAGALSGAFASVATMPFDVGKTRTQVYRDTGAATKAAIAVTM
FG09323.1 	RRRSQAQENHTETFVDAFTAGALSGAFASFVTTPFDVGKTRTQIYQGTSKKAKQSGG---
AN6087.1  	STQLGTSDVEGNTFLDSFVAGSISGALAALVTTPFDVGKTRQQVFRHMGDDAPSTAGSKL
          	  :  : : . :** *:* **: ***:*:..* ******** *:::  .  :  : . . 

MG03631.1 	---AIAPEERSMVQLLWHIFRTEGAAGLFKGWIPRTLKVAPACAIMISSYEVGKRSFRSM
NCU07478.1	EKSAVRPEERNMARLLWHIFSTEGVAGLFRGWIPRTLKVAPACAIMISSYEVGKRAFRGM
FG09323.1 	--STAAPEQRSMVRLLWHIFKTEGASGLWKGWIPRTLKVAPACAIMISSYEVGKRAFRGV
AN6087.1  	PKGPLQPEQLSLPKFLLHIFREEGAAGLFRGWVARCLKVAPACAIMISTYEVGKKFARDV
          	 ...  **: .: ::* ***  **.:**::**:.* ************:*****:  *.:

MG03631.1 	NERK-LRQEMELKQS-----------
NCU07478.1	NERAAMKEREELAQQGQQQEQSDEEA
FG09323.1 	NERQ-LNGSTSTGQSS----------
AN6087.1  	NERRQSPPDGNVSDAS----------
          	***       .  : .
```
